# Validation of the German version of the STarT-Back Tool (STarT-G): a cohort study with patients from primary care practices

**DOI:** 10.1186/s12891-015-0806-9

**Published:** 2015-11-11

**Authors:** Sven Karstens, Katja Krug, Jonathan C. Hill, Christian Stock, Jost Steinhaeuser, Joachim Szecsenyi, Stefanie Joos

**Affiliations:** Department of General Practice and Health Services Research, University Hospital Heidelberg, Heidelberg, Germany; Institute of Primary Care and Health Sciences, Keele University, Keele/Stoke-on-Trent, United Kingdom; Institute of Medical Biometry and Informatics, University of Heidelberg, Heidelberg, Germany; Institute of Family Medicine, University Hospital Schleswig-Holstein Campus Luebeck, Luebeck, Germany; Institute for General Practice and Interprofessional Care, University Hospital Tuebingen, Tuebingen, Germany

**Keywords:** Low back pain, Questionnaire, Psychosocial factors, Therapy prognosis

## Abstract

**Background:**

Current research emphasizes the high prevalence and costs of low back pain (LBP). The STarT Back Tool was designed to support primary care decision making for treatment by helping to determine the treatment prognosis of patients with non-specific low back pain. The German version is the STarT-G. The cross-cultural translation of the tool followed a structured and widely accepted process but to date it was only partially validated with a small sample.

The aim of the study was to test the psychometric properties construct validity, discriminative ability, internal consistency and test-retest-reliability of the STarT-G and to compare them with values given for the original English version.

**Methods:**

A consecutive cohort study with a two-week retest was conducted among patients with non-specific LBP, aged 18 to 60 years, from primary care practices. Questionnaires were collected before the first consultation, and two weeks later by post, using the following reference standards: the Roland and Morris disability questionnaire, the Tampa Scale of Kinesiophobia, the Pain Catastrophizing Scale and the Hospital Anxiety and Depression Scale. Psychometric properties examined included the tool’s discriminative abilities, whether the psychosocial subscale was one factor, internal consistency, item redundancy, test-retest reliability and floor and ceiling effects.

**Results:**

There were 228 patients recruited with a mean age of 42.2 (SD 11.0) years, and 53 % were female. The areas under the curve (AUC) for discriminative ability ranged from 0.70 (STarT-G Subscale - Pain Catastrophizing Scale; CI95 0.63, 0.78) to 0.77 (STarT-G Total - Composite reference standard, CI95 0.60, 0.94). Factor loadings ranged from 0.49 to 0.74. Cronbach’s alpha testing the internal consistency and redundancy for the total/subscale scores were α = 0.52/0.55 respectively. The STarT-G test-retest reliability Kappa values for the total/subscale scores were 0.67/0.68 respectively. No floor or ceiling effects were present.

**Conclusions:**

The STarT-G shows acceptable psychometric properties although not in exact agreement with the original English version. The items previously regarded as a psychosocial subscale may be better seen as an index of different individual psychosocial constructs. The relevance of using the tool at the point of consultation should be further examined.

## Background

Low back pain (LBP) ranks the highest of all conditions in terms of years lived with disability, both in Western Europe and Worldwide [1]. This has enormous economic consequences on health care expenditure and loss of work productivity [2, 3]. Complex biopsychosocial mechanisms are known to be involved in the development of chronic disabling LBP and as a result the variation in treatment prognosis for individual patients is considerable [4]. This means, that although a number of effective treatments are available, selecting appropriate treatment for individual patients remains a substantial clinical challenge for primary care where most patients are managed [5–7].

An internationally agreed research priority is to test whether the effectiveness of treatment results might be improved by early identification of patient-subgroups that are most at risk for developing chronic disabling pain [8]. Several different approaches for subgrouping patients with low back pain have been developed [9]. One with feasibility and demonstrated clinical and cost effectiveness is the STarT Back stratified care approach involving the use of the Keele STarT Back Tool (SBT), which has specifically been designed for primary care to fast-track patients to appropriate treatment based on prognostic information. The tool assists clinicians in determining an individual’s likely prognosis and consists of nine items, with the first four relating to biomedical factors and the last five identifying modifiable psychosocial risk factors [10]. Patients are then allocated to one of three prognostic groups (low, medium and high risk) using established scoring cut-offs which each have different matched treatment recommendations [11, 12]. Patients at low risk receive support to self-manage and are deliberately not over treated or investigated. Patients at medium risk are provided with evidence-based physiotherapy treatment such as exercise and manual therapy, and patients at high risk receive psychologically informed physiotherapy, which integrates physical and psychological approaches, aiming at reducing obstacles to recovery such as unhelpful beliefs and illness behaviors. Following promising research findings the approach is gradually being implemented into routine practice in the UK [13] and some initial research has reported promising findings that implementing this approach in Germany might be possible [14].

The SBT was originally developed in the UK and has been translated into over 25 languages with 15 articles validating the psychometric properties of different versions [15]. The German version (STarT-G) was developed following a structured and widely accepted cross-cultural translation process [16]. In addition, some initial psychometric testing on a small cohort in Switzerland has been reported [17]. However, as the sample size was small and only a limited number of measurement properties were tested, we aimed to conduct a larger scale German validation study.

The specific objectives of our study were to test the STarT-G’s construct validity, discriminative ability, internal consistency and test-retest-reliability and to compare findings with the values given for the original English version.

## Methods

A cohort study with a two-week retest has been conducted. Patients with LBP were recruited from primary care (general practices and orthopaedic practices both with free access to care). The first questionnaire (t0) took place within the practice before consultation, and the second questionnaire was sent by post (t1) 10 days later. Patients who did not respond to the postal questionnaire were contacted by telephone.

Nine general practices and two orthopaedic practices participated with eight being single-handed, and three having more than one physician. Before patients were recruited, each practice received training from the study-team involving:

- An introduction to the STarT Back Tool,
- The informed consent procedure,
- Information about the data collection procedure,
- Information on transferring collected data from the practice to the study center,
- Information about study reimbursement.

Inclusion criteria were patients with non-specific LBP, aged 18 to 60 years. The diagnosis of low back pain was defined as being specific, if a patient had a cauda equina syndrome, an inflammatory disorder such as ankylosing spondylitis, or had a suspected serious pathology such as a tumor or vertebral fracture. No restrictions were placed on the duration of a patient’s back pain symptoms. Patients were excluded, if they had consulted the physician within the last twelve weeks, had undergone spinal surgery within the last six months, or if they were unable to complete the study questionnaires due to poor German language skills. Anonymized information on eligible patients’ age and gender was obtained regardless of study participation (“consent list”).

The retest-material was sent to patients 10 days after the baseline assessment from the study-center. This duration was set to counter memory effects. Since it was likely that the health status would change at least for a part of the patients, an additional question on the subjective estimation of whether their complaints had changed over this period, was added [18]. Patients who did not respond to the postal questionnaire within two weeks were telephoned and reminded to send the questionnaire, or alternatively asked to answer a limited set of questions. The retest-process was managed by a specifically constructed database to ensure the maintenance of the predefined time intervals.

Ethical approval was granted by the Ethics Committee of the University of Heidelberg (registration ID: S-414/2013). All patients gave their written informed consent for participation before entering the study in the participating practice.

### Instruments

In addition to the STarT-G, several validated German versions of reference standard instruments were included in the study questionnaire. Disability was operationalized using the Roland and Morris disability questionnaire (RMDQ) [19], fear avoidance beliefs were operationalized with the 17-item-version of the Tampa Scale of Kinesiophobia (TSK) [20], catastrophizing with the Pain Catastrophizing Scale (PCS) [21] and depression with the Hospital Anxiety and Depression Scale (HADS) [22]. Pain intensity was measured using the mean of three eleven-point box-scales for least, average (over the previous two weeks), and current pain [23, 24]. Standardized questions were used for documentation of the patients’ age, gender and body-mass-index (BMI), information on type of employment, days off work due to LBP and the duration of the back pain episode [25, 26].

The wording of two questions of the STarT-G were slightly modified lowering their item-difficulty. Because of the very high difficulty of item 5 and 8 found within the first study conducted in Switzerland, a rewording was undertaken in agreement with the developers of the SBT [17]. The STarT-G can be obtained from the authors via email.

The definitions for reference standard cases were catastrophizing (PCS score ≥ 20), fear (TSK score ≥ 41), depression (HADS-D score ≥ 8) and disability (RMDQ score ≥ 7) [11, 22]. Furthermore, a composite reference standard (CRS; ‘distress’) was determined, defined by individuals that were a ‘case’ simultaneously in the three psychosocial reference standard questionnaires: TSK, PCS and HADS depression. Following pretesting with selected LBP patients, the estimated time for the entire study questionnaire completion was 15 minutes.

### Statistical analyses

Descriptive statistics were calculated to characterize the study population. The baseline characteristics of study participants were described to allow interpretability of the study sample, together with data about drop-outs, missing data and recruitment rate.

Discriminative ability was assessed by computing receiver operating characteristic curves with areas under the curves (AUC) and 95 % confidence interval (CI). Consistent with the original validation of the English SBT, this was done for disability, catastrophising and distress [27]. Adjectives that can be used to describe AUC-values have been proposed by Hosmer and Lemeshow with an AUC = 0.5 suggesting ‘no discrimination’, 0.7 to < 0.8 considered ‘acceptable discrimination’, 0.8 to 0.9 considered ‘excellent discrimination’ and >0.9 considered ‘outstanding discrimination’ [28]. To determine if a patient was a ‘case’ on reference standard instruments, the individual’s scores were compared to cut-off values given under the subheading Instruments (see “definitions for reference standard cases”). Since the CI determined by Hill et al. did not fall short of AUC = 0.7 [10], equivalence was expected if the lower CI did not fall short of the same cut-off.

In addition to the AUC, helping to interpret the relations between the instruments, Spearmans correlation coefficients were calculated for the STarT-G total and subscale scores for the RMDQ, TSK, PCS and HADS depression scores in order to be consistent to the approach of the original SBT authors.

To test if the psychosocial subscale could be regarded as one factor, a principal components analysis was undertaken. In general, at least four items should exceed 0.6 [29]. For the original version of the SBT, factor loadings between 0.6 and 0.8 were calculated; therefore equivalence was expected if the STarT-G values would exceed 0.6 for these five psychosocial items.

To determine internal consistency and item redundancy for the psychosocial subscale, the Cronbach’s alpha was calculated (poor internal consistency was defined as α < 0.70, item redundancy was defined as α > 0.90) [30]. Since the original SBT validation study reported values ranging between 0.7 and 0.9, equivalence was expected if Alpha was within this same range.

To investigate the test-retest reliability, Cohen’s quadratic weighted Kappa was calculated for the overall and subscale scores [31]. Since we had to expect that the health status would change between t0 and t1 at least for some patients, and that the STarT-G is responsive, test-retest calculations were limited to patients who self-reported their health problems to be unchanged over the two time-points [32]. A range between Kappa 0.6 and 0.8 was defined as good agreement. The values of 0.79 for the SBT total score and 0.76 for the subscale score calculated by Hill et al. lay within this range [10]. Therefore, equivalence was expected with a Kappa score of > 0.6. A sensitivity analysis was planned excluding retest data gathered via telephone.

Floor and ceiling effects were defined as present if more than 15 % of the responders achieved the lowest or highest possible STarT-G total score [33].

All statistical tests were two-sided and a significance level of alpha = 5 % was used. Analysis was generally performed using SPSS version 20.0. Principal component analyses and Kappa calculations were performed using the R language and environment for statistical computing, version 3.1.1 [34].

### Sample size

Principal component analysis was expected to be the procedure with the need for the largest sample size. For calculation, the formula given by Bortz and Schuster was considered [29]. With a minimally expected factor loading of 0.4 and a stability of 0.9, a sample size of *n* = 180 resulted. This led to the conclusion that using the same sample size of 200 as defined for the original SBT validation study would be sufficient [10].

## Results

Consent for participation was given by 228 patients (90.1 %), with 25 declining (9.9 %). Consenters and non-consenters did not differ statistically significant by age or gender. The mean age of study participants was 42.2 (SD 11.0) years, and 53 % were female (Table 1). During the previous twelve weeks before t0, 31 patients (13.6 %) reported having taken some sick leave, with a mean of 13.3 (SD 21.2) days off work. The t1 questionnaire was returned by 181 patients (79.4 %), with an additional 4 patients answering questions on the telephone (1.8 %; 81.1 % in total). Non-respondents at t1 were significantly younger and more often male. The mean time difference between the completion of the t0 and the t1 questionnaire was 21.1 (SD 13.3) days.

**Table 1:** Characteristics of the study population, *n* = 228

Mean age in years (SD) n	42.2 (11.0)	228
Gender female %, n	53	120
Mean Body-Mass-Index in kg/m^2^ (SD) n	26.7 (5.0)	225
Employment %, n		
Not working	4.9	11
Working ≥ 35 hours	62.9	141
Working 15 to 34 hours	25.0	56
Working < 15 hours	2.7	6
Parental or other leave	3.1	7
Trainee/retrainee/prentice	1.3	3
Total	100	224
Mean Pain intensity (SD) n	4.3 (1.7)	225
Mean Disability (RMDQ; SD) n	9.9 (5.2)	204
Duration of current episode %, n		
<6 weeks	60.3	135
6 to 12 weeks	8.9	20
>12 weeks to 0.5 year	5.8	13
>0.5 year	25.0	56
Total	100	224
Sick leave within the previous 12 weeks %, n^a^	13.6	31
Mean HADS-D (depression; SD) n	4.7 (3.5)	224
Mean PCS (SD) n	16.7 (10.5)	227
Mean TSK (SD) n	32.9 (6.7)	228
Mean STarT-G total (SD) n	3.9 (2.0)	205
Mean STarT-G subscale (SD) n	2.0 (1.4)	209
STarT-G risk group %, n		
Low risk	38.6	80
Medium risk	43.5	90
High risk	17.9	37
Total	100	207

*HADS-D* Hospital Anxiety and Depression Scale (Depression), *PCS* Pain Catastrophizing Scale, *RMDQ* Roland Morris Disability Questionnaire, *TSK* Tampa Scale of Kinesiophobia

^a^Medically certified sick within twelve weeks before t0

Due to missing answers (9 questionnaires) or invalid answers (12 questionnaires, both (missing and invalid answers) 2 questionnaires), it was not possible to calculate the STarT-G total score for 23 patients and subscale score for 19 patients.

**Fig. 1:**
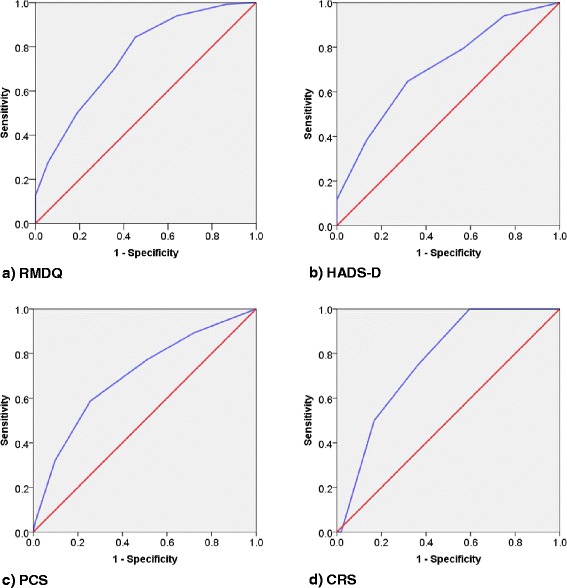
**a**–**d** Receiver operating characteristic curves. HADS-D: Hospital Anxiety and Depression Scale (Depression), PCS: Pain Catastrophizing Scale, RMDQ: Roland Morris Disability Questionnaire, CRS: composite reference standard

The AUCs for STarT-G’s ability to discriminate reference standard cases ranged from 0.70 to 0.77, indicating acceptable discrimination (Table 2 and Fig. 1a–d). Since the lower CIs all fell short of 0.7, the STarT-G’s discriminative abilities were not equivalent to the original SBT version according to our pre-defined criteria.

**Table 2:** Areas under the curve (AUC)

	AUC	SE	95 % CI	
STarT-G Total - RMDQ	0.76	0.04	0.68	0.83
STarT-G Sub - PCS	0.70	0.04	0.63	0.78
STarT-G Sub - HADS-D	0.71	0.05	0.61	0.81
STarT-G Sub - CRS	0.77	0.09	0.60	0.94

HADS-D: Hospital Anxiety and Depression Scale (Depression), PCS: Pain Catastrophizing Scale, RMDQ: Roland Morris Disability Questionnaire, CRS: composite reference standard

All correlation coefficients were significant (Table 3), with values ranging from 0.28 to 0.46.

**Table 3:** Spearman correlation coefficients, for each instrument for STarT-G-total score and subscale score

	Total	95 % CI		Subscale	95 % CI	
RMDQ	0.46^a^	0.32	0.58	0.39^a^	0.25	0.52
PCS	0.30^a^	0.17	0.43	0.40^a^	0.27	0.52
TSK	0.28^a^	0.14	0.41	0.30^a^	0.17	0.42
HADS-D	0.32^a^	0.19	0.43	0.34^a^	0.21	0.46

*HADS-D* Hospital Anxiety and Depression Scale (Depression), *PCS* Pain Catastrophizing Scale, *RMDQ* Roland Morris Disability Questionnaire, *TSK* Tampa Scale of Kinesiophobia

^a^significant (two-tailed, level = 0.01)

Factor loadings ranged from 0.49 to 0.74 with items 5 and 6 falling short of the predefined threshold (<0.6) for equivalence with the original SBT (Table 4).

**Table 4:** Correlation of the variables with the component

STarT-G	PC1	h^2^	u^2^
Item 5	0.49	0.24	0.76
Item 6	0.51	0.26	0.74
Item 7	0.74	0.55	0.45
Item 8	0.63	0.39	0.61
Item 9	0.65	0.42	0.58

With α = 0.55 for the subscale score, and α = 0.52 for the total score, internal consistency was poor. Since Cronbach’s alpha fell short of the predefined threshold for equivalence, equivalence to the original version of the SBT was not reached.

Data from 30 patients, who self-rated their health problems as unchanged, was included in the analysis for the test-retest-reliability. With Kappa = 0.67/0.68 (95 % CI 0.46-0.81/0.48-0.84) for the total score and the subscale score respectively, agreement was good and equivalent to the original version of the SBT. An additional calculation for the risk-groups resulted in Kappa = 0.28 (95 % CI 0.23-0.55). The planned sensitivity analysis excluding retest data gathered via telephone was not possible as none of the four patients responding via telephone rated their health problems as unchanged.

With equivalence to the original SBT version, no floor or ceiling effects were found for the STarT-G (3.9 % (*n* = 8) patients with 0 points; no patients with 9 points).

## Discussion

The STarT-G did not show identical psychometric properties to the original English version although our risk-group distribution was comparable to the original SBT validation study. The measurement properties of the STarT-G showed good test-retest reliability for the total score and the subscale score, acceptable discriminative ability and no floor or ceiling effects. Correlations with the total score reference standards for disability, and subscale score for catastrophising were acceptable. The correlations with the subscale score reference standards for kinesiophobia and depression and the test-retest reliability for risk-group allocation among patients who reported stable symptoms were weak. The psychosocial subscale of the STarT-G (items five to nine) should not be regarded as one distress factor, as the internal consistency of the corresponding items was poor.

According to pre-defined criteria the properties of the STarT-G differ in a number of respects from those of the original SBT. It is common that measurement tools perform less well in further validation studies in comparison to the developmental study [35]. In addition, we were not able to identify commonly accepted methods for the comparison of the measurement properties of the two instrument versions. A method we found to compare the tools’ discriminative abilities has been subject to criticism [27]. Therefore we used a pragmatic approach, which may have led to an over-estimation of the differences between both instruments. Nevertheless, international comparisons between different cohorts using the STarT-G and the SBT should be undertaken cautiously.

Our decision to exclude patients who had consulted their physician within the previous twelve weeks meant that in comparison to the original study sample and other SBT related studies we had greater numbers of patients with a short duration back pain symptoms [36–41]. As a result, some of the differences in the STarT-G’s psychometric properties may be due to the differences between the populations studied. The results of Beneciuk et al. support this perspective, as they specifically analyzed the relevance of the timing of the SBT and found that especially in acute high-risk patients, tool scores often change within a few weeks. They therefore suggest further research examining the potential of repeated measures in helping to improve the accuracy of prognostic assessment in this subgroup [42].

In the original SBT validation study the psychosocial subscale was confirmed to be one overall ‘distress’ factor. However, this finding was not replicated in our STarT-G validation study and in fact is consistent with a Danish validation study which described the SBT as ‘a multidimensional questionnaire consisting of one or two screening questions for each of eight underlying constructs’ and therefore recommended a stronger item-based approach for validation [36]. Whilst this finding is of interest, it might not have a large impact on the STarT-G’s ability of stratify patients in primary care.

In relation to relevant reference standards our results identified that the psychosocial subscale of the STarT-G and the TSK (for fear avoidance beliefs) had the lowest correlation. The reason for this issue is unclear although it is noted that in an updated version of the TSK, the instrument includes six items less than the version we used. This reduction was triggered by problems described for the TSK factor-structure in the international literature [20]. Therefore, in future studies it might be useful to include other instruments for fear avoidance or other versions of the TSK. For the study described in this manuscript we slightly modified the wording of two previous STarT-G items, number five about fear of movement (kinesiophobia) and number eight about low mood (depression). It is possible that the lower correlation between the subscale score and reference standards for kinesiophobia and depression was due to this re-wording, however this would seem unlikely, especially since the new formulation of item five resulted in a higher conformity to the original TSK-item which was used to develop the STarT-Tool [8].

Missing answers leading to invalid STarT-G scores - although anticipated - were only a small proportion (<4 %), with more than half of these due to patients giving ambiguous responses, e.g. ticks in between the ‘Agree’/’Disagree’ boxes. For the Chinese version of the SBT missing data was also an issue although to a lesser extent [41]. Our results suggest that an estimated tenth of all patients return incomplete STarT-scores making it impossible to calculate their risk subgroup without further enquiry. This occurred despite specific attention from the study team to ensure appropriate instructions were in bold, such as ‘answer each question if possible and try not to spend too long over your answers but pick the answer that first comes to mind’. In clinical practice therapists clearly have the opportunity to ask patients if this occurs. However, we recommend that a clear definition and evaluation method of missing-data for the SBT is considered in order that more responses with missing data are able to be used.

In Germany, it is likely that physiotherapists and primary care physicians will be the primary users of the STarT-G [13]. This study, provides them with clear information on the instrument’s measurement properties. The stratification tool is designed to assist clinicians in their decision-making process and not to replace their decision-making. In addition, the STarT Back approach upskills physiotherapists, through a training course, to address the complex needs of high-risk patients through the delivery of ‘psychologically informed physiotherapy’. Evidence suggests that trained physiotherapists are effective in managing around 85 % of this high-risk complex patient subgroup [43–46]. Nevertheless, the systematic review by Kenny et al. concludes that, from an international perspective, the SBT should still be considered as being in a developmental stage and should therefore be used with caution in practice [15]. Our results indicate that this also applies for the German version.

Strengths of this study were that the design was clearly planned *a priori* and rigorously applied. Experiences from a first small validation pilot in Switzerland reinforced the selection of primary care practices as the appropriate setting for this study since the clinical context has a strong influence on the answers patients provide. Moreover, in contrast to the Swiss study, pre first-contact consultation as the first time-point for administrating the STarT-G was chosen to include patients who had not yet received any treatment [17, 47]. In addition, a half-year mailing is currently being carried out, to determine the predictive ability of the STarT-G. Other strengths include achieving an appropriate sample size and ensuring non-responders were contacted by telephone to ensure a high follow-up rate. In respect to interpreting the test-retest-reliability findings some caution is appropriate, since the Kappa-value calculations were limited to a relatively small subsample of patients who self-reported their health problems as being unchanged for the observed study period.

The potential for cost savings and better cost-effectiveness of the STarT-approach has been demonstrated by Hill et al. in a large randomized controlled trial [11] and is also supported by the results from a prospective population-based sequential comparison observing the implementation of the STarT-Back-Approach in routine health care [13]. Nevertheless, results have yet to be replicated in other countries. Foster et al. propose that randomized trials are the preferred optimal design for this [9]. Correspondingly, the next step should be a feasibility pilot to examine the approach in Germany and provide information to help develop a clear implementation strategy. We have therefore conducted a related qualitative study with GPs and physiotherapists to compliment this research [14].

## Conclusion

The STarT-G shows overall acceptable psychometric properties, although some differences with the original English version were identified. These included the items previously regarded as a psychosocial subscale being found to be more than one construct, and so we recommend this subscale is better understood to be a collection of individual psychosocial items. Further research utilizing the STarT-G should consider our findings and pay attention to establishing methods to deal with missing values.

## Abbreviations

AUC: Area under the curve
BMI: Body-mass-index
CRS: Composite reference standard
HADS-D: Hospital anxiety and depression scale (depression)
LBP: Low back pain
PCS: Pain catastrophizing scale
RMDQ: Roland and Morris disability questionnaire
SBT: STarT back tool
STarT: Subgroups for targeted treatment
STarT-G: German version of the STarT Back Tool
TSK: Tampa scale of kinesiophobia
